# Fracture resistance in endodontically treated teeth restored with custom-milled zirconia versus prefabricated fiber posts: An *in vitro* study

**DOI:** 10.6026/973206300211865

**Published:** 2025-07-31

**Authors:** Ojaswini Pawar, Jasmine Marwaha, Deepankar Dass, Vipin Arora, Ashtha Arya, Akansha Tilokani

**Affiliations:** 1 Department of Conservative Dentistry and Endodontics, D.Y. Patil Dental School, Charholi Budruk, Lohegaon, Pune, India; 2 Department of Conservative dentistry and Endodontics, National Dental College and Hospital, Derabassi-140507, Punjab, India; 3 Department of Conservative Dentistry & Endodontics, Maharshi Markendshwar College of Dental Sciences & Research, Mullana, Haryana, India; 4 Department of Restorative Dental Sciences, Faculty of Dentistry, Taif University, Kingdom of Saudi Arabia; 5 Department of Conservative Dentistry and Endodontics, SGT Dental College, Hospital and Research Institute, SGT University, Gurugram, Haryana- 122505, India; 6 Department of conservative dentistry and endodontics, Kalinga institute of dental sciences, KIIT deemed to be university, Bhubaneswar, Odisha, India

**Keywords:** Endodontics, post-and-core, zirconia, fiber posts, fracture resistance, CAD-CAM

## Abstract

The resistance to fracture of endodontically-treated maxillary central incisors restored using the custom-milled zirconia, post group, versus the prefabricated fiber post group is of interest. Hence, sixty teeth were separated into two groups and restored similarly and undergo static loading. Fracture resistance of zirconia post was significantly greater (809 45 19 NY 8219 on average) than fiber post (567 26 19 NY 821). The main outcomes in the case of zirconia posts were the non-resettable failures (83 per cent), but in fiber posts more of the outcomes were resettable (73 per cent). Thus, post selection shows trade-off with mechanical strength and ability to repair.

## Background:

Endodontically treated teeth present unique restorative challenges due to the structural alterations resulting from access cavity preparation, canal instrumentation and loss of pulpal vitality [1]. These factors contribute to increased susceptibility to fracture, with vertical root fractures representing one of the most serious complications in endodontic therapy [2]. The restoration of extensively damaged endodontically treated teeth often requires the placement of intraradicular posts to provide retention for coronal restorations and to reinforce the remaining tooth structure. The selection of appropriate post materials and designs has evolved significantly over the past decades. Traditional cast metal posts, while providing excellent mechanical properties, have been associated with unfavourable stress distribution patterns and catastrophic root fractures due to their high elastic modulus [3]. This has led to the development of alternative post systems, including prefabricated fiber posts and more recently, custom-milled ceramic posts. Prefabricated fiber posts have gained widespread acceptance due to their elastic modulus similarity to dentin (approximately 20 GPa), which theoretically provides more favorable stress distribution and reduces the risk of root fracture [1]. Concerns remain regarding their fracture resistance under high loading conditions, particularly in posterior teeth or patients with parafunctional habits [4, 5]. The advent of computer-aided design and computer-aided manufacturing (CAD-CAM) technology has enabled the fabrication of custom-milled posts from various materials, including zirconia [6]. Zirconia offers excellent biocompatibility, superior aesthetics and exceptional mechanical properties, with flexural strength values ranging from 900-1200 MPa. Custom-milled zirconia posts can be precisely fitted to the canal anatomy, potentially optimizing retention and stress distribution [7]. This mechanical mismatch may lead to the development of microcracks and eventual catastrophic failure, particularly under cyclic loading conditions. However, the superior mechanical properties of zirconia may provide advantages in high-stress environments where maximum fracture resistance is required [8, 9, 10- 11]. The mode of failure represents a critical clinical consideration in post selection. Restorable failures that occur above the alveolar crest allow for retreatment and preservation of the tooth, while catastrophic failures involving the root structure typically necessitate extraction. Understanding the relationship between post material properties and failure modes is essential for evidence-based clinical decision-making. Despite the growing interest in custom-milled zirconia posts, comprehensive comparative studies evaluating their performance against established fiber post systems remain limited. Furthermore, the impact of CAD-CAM manufacturing on post fit and clinical performance requires further investigation [12]. Therefore, it is of interest to address these knowledge gaps by comparing the fracture resistance and failure modes of custom-milled zirconia posts with prefabricated fiber posts in a controlled laboratory setting.

## Materials and Methods:

## Specimen preparation:

Sixty freshly extracted human maxillary central incisors were collected following approval from the institutional ethics committee (Protocol #2023-ETH-45). Teeth were selected based on specific inclusion criteria: intact crown and root structure, absence of caries or previous endodontic treatment, root length between 12-15 mm and similar buccolingual and mesiodistal dimensions. Specimens with visible cracks, calcifications, or anatomical irregularities were excluded from the study. Following extraction, soft tissue remnants and calculus were mechanically removed and teeth were stored in 0.1% thymol solution at 4°C for no more than three months prior to testing. All specimens were decoronated 2 mm coronal to the cementoenamel junction using a diamond disc under water cooling to simulate extensive coronal destruction requiring post-core restoration.

## Endodontic treatment:

Standard endodontic treatment was performed on all specimens using the crown-down technique with nickel-titanium rotary instruments (ProTaper Universal, Dentsply Sirona, Charlotte, NC, USA). Root canals were irrigated with 2.5% sodium hypochlorite and 17% EDTA, followed by final irrigation with sterile saline. Working length was established 1 mm short of the apical foramen using electronic apex locators (Root ZX, J. Morita Corp., Tokyo, Japan). Root canals were dried with sterile paper points and obturated using the warm vertical compaction technique with gutta-percha and AH Plus sealer (Dentsply Sirona, Charlotte, NC, USA). Access cavities were temporized with glass ionomer cement (Fuji IX, GC Corporation, Tokyo, Japan) and specimens were stored in 100% humidity at 37°C for 72 hours to allow complete sealer setting.

## Post space preparation:

Post space preparation was initiated 48 hours after obturation using Gates-Glidden burs and Peeso reamers (Dentsply Sirona, Charlotte, NC, USA). Post length was standardized at 10 mm, corresponding to approximately two-thirds of the root length. Post space diameter was prepared to 1.5 mm, maintaining a minimum of 4 mm of apical gutta-percha seal. Canal walls were refined using diamond-coated burs to ensure uniform taper and smooth surface finish.

## Group allocation and post fabrication:

Specimens were randomly allocated into two groups using computer-generated randomization (n=30 each):

## Group A (Custom-milled zirconia posts):

Post space impressions were made using polyvinyl siloxane impression material (Express XT, 3M ESPE, St. Paul, MN, USA). Impressions were scanned using a high-resolution laboratory scanner (Ceramill Map 400, Amann Girrbach and Koblach, Austria) and custom zirconia posts were designed using CAD software (Ceramill Mind, Amann Girrbach) with standardized core dimensions [12]. Posts were milled from pre-sintered zirconia blocks (IPS e.max ZirCAD, Ivoclar Vivadent, Schaan, Liechtenstein) using a 5-axis milling machine (Ceramill Motion 2, Amann Girrbach) and sintered according to manufacturer specifications.

## Group B (Prefabricated fiber posts):

Glass fiber posts (RelyX Fiber Post, 3M ESPE, St. Paul, MN, USA) were selected to match the prepared post space diameter. Posts were tried in to ensure passive fit with approximately 2 mm extending coronally from the canal orifice. Composite cores were built using dual-cure resin composite (RelyX U200, 3M ESPE) around the coronal portion of the fiber posts using standardized silicone matrices to ensure uniform core dimensions.

## Post cementation:

All post spaces were cleaned with 37% phosphoric acid for 15 seconds rinsed and dried with paper points. Custom-milled zirconia posts were sandblasted with 50 µm aluminium oxide particles and silanized (RelyX Ceramic Primer, 3M ESPE). All posts were cemented using dual-cure resin cement (RelyX U200, 3M ESPE) according to manufacturer instructions. Excess cement was removed and specimens were light-cured for 40 seconds from multiple directions using a LED curing unit (Elipar DeepCure-S, 3M ESPE) with light intensity of 1470 mW/cm^2^.

## Crown fabrication and cementation:

Standardized porcelain-fused-to-metal (PFM) crowns were fabricated for all specimens using a master die technique to ensure dimensional consistency. Crown preparations were completed with 1.5 mm cervical shoulder finish lines and 2 mm occlusal reduction. Impressions were made using polyvinyl siloxane impression material and crowns were fabricated with base metal alloy substructures (Wiron 99, BEGO and Bremen, Germany) veneered with feldspathic porcelain (VITA VM 13, VITA Zahnfabrik, Bad Säckingen, Germany). All crowns were cemented using glass ionomer cement (RelyX Luting, 3M ESPE) under standardized seating pressure (5 kg for 10 minutes). Excess cement was removed and specimens were stored in distilled water at 37°C for 24 hours before thermocycling.

## Thermocycling:

All specimens underwent thermocycling (5000 cycles between 5°C and 55°C with 30-second dwell times and 5-second transfer times) using an automated thermocycling machine (Thermocycler THE-1100, SD Mechatronik, Feldkirchen-Westerham, Germany) to simulate oral temperature variations and aging effects.

## Fracture resistance testing:

Specimens were embedded in self-curing acrylic resin (Palapress Vario, Heraeus Kulzer, Hanau, Germany) to 2 mm apical to the crown margins, simulating alveolar bone support. Fracture resistance testing was performed using a universal testing machine (Instron 3345, Instron Corporation, Norwood, MA, USA) with a custom jig designed to apply load at 135° to the long axis of the tooth, simulating clinical loading conditions [1, 7]. A spherical steel indenter (diameter 5 mm) was positioned on the lingual surface of the crown, 2 mm from the incisal edge. Load was applied at a constant crosshead speed of 1.0 mm/min until catastrophic failure, defined as the first major drop in load-displacement curve exceeding 25% of maximum load. Maximum fracture load values were recorded in Newtons.

## Failure mode analysis:

Fractured specimens were examined under stereomicroscope (Leica M125, Leica Microsystems, Wetzlar, Germany) at 16 x magnifications to classify failure modes. Failures were categorized as:

[1] Type I (Favorable/Restorable): Cohesive failure within post or core material, or adhesive failure at post-core interface, occurring coronal to the cervical finish line

[2] Type II (Unfavorable/Non-restorable): Root fracture extending apical to the cervical finish line, requiring extraction

Representative specimens from each failure mode were further analyzed using scanning electron microscopy (SEM) (JSM-6490LV, JEOL Ltd., Tokyo, Japan) to characterize fracture surfaces and failure mechanisms.

## Statistical analysis:

Sample size calculation was performed using G*Power software (version 3.1.9.7) based on pilot study data, indicating that 30 specimens per group would provide 80% power to detect a clinically significant difference of 150 N in fracture resistance with α=0.05. Data normality was assessed using Shapiro-Wilk tests. Fracture resistance values were compared between groups using independent t-tests. Failure mode distributions were analyzed using chi-square tests. All statistical analyses were performed using SPSS software (version 28.0, IBM Corporation, Armonk, NY, USA) with significance level set at α=0.05.

## Results:

All 60 specimens successfully completed the experimental protocol without premature failure during preparation or thermocycling phases. Shapiro-Wilk tests confirmed normal distribution of fracture resistance data for both groups (p>0.05). The mean fracture resistance values and descriptive statistics are presented in Table 1 (see PDF). Custom-milled zirconia posts (Group A) demonstrated significantly higher fracture resistance (809.3 ± 45.2 N) compared to prefabricated fiber posts (Group B) (567.5 ± 26.6 N) (p<0.001, 95% CI: 216.7-266.9 N). The difference represents a 42.6% increase in fracture resistance for zirconia posts compared to fiber posts. Significant differences were observed in failure mode distribution between groups (χ^2^ = 18.76, p<0.001). The detailed failure mode analysis is presented in Table 2 (see PDF).

## Group A (Custom-milled zirconia posts):

The majority of failures (83.3%) were classified as Type II (non-restorable), characterized by vertical root fractures extending from the cervical region to the middle or apical third of the root. SEM analysis revealed that failures typically initiated at the post-dentin interface due to stress concentration, propagating through the root structure in a predominantly vertical pattern. Five specimens (16.7%) exhibited Type I failures involving fracture of the zirconia post itself or debonding at the post-core interface.

## Group B (Prefabricated fiber posts):

Type I (restorable) failures predominated (73.3%), typically involving cohesive fracture within the composite core material or adhesive failure at the post-core interface. These failure occurred coronal to the finish line, preserving root structure integrity. Eight specimens (26.7%) showed Type II failures, characterized by oblique root fractures, generally less extensive than those observed in the zirconia group. Analysis of load-displacement curves revealed distinct failure behaviors between groups. Zirconia posts exhibited relatively linear elastic deformation followed by sudden catastrophic failure with minimal plastic deformation. In contrast, fiber posts demonstrated more gradual failure progression with evidence of plastic deformation before ultimate failure, consistent with their lower elastic modulus and ability to absorb energy during loading.

The fracture resistance values obtained in this study align with recent investigations comparing similar post systems. The zirconia post fracture resistance (809 N) falls within the range reported by previous studies (435-809 N) [1, 6], while fiber post values (567 N) are consistent with literature reports (342-916 N) [1, 2 and 5]. The observed failure mode distributions similarly correspond to previous findings demonstrating higher incidence of catastrophic failures in rigid post systems [1, 2].

## Discussion:

The results of this study provide important insights into the comparative performance of custom-milled zirconia posts and prefabricated fiber posts in endodontically treated teeth. The significant difference in fracture resistance (42.6% higher for zirconia posts) confirms the superior mechanical properties of zirconia material, while the contrasting failure mode patterns highlight the clinical implications of material selection in post-endodontic restoration. The superior fracture resistance demonstrated by custom-milled zirconia posts can be attributed to several factors. Zirconia's exceptional mechanical properties, including flexural strength of 900-1200 MPa and fracture toughness of 6-10 MPa.m 1/2, provide inherent advantages over fiber-reinforced composite materials [1]. The CAD-CAM manufacturing process enables precise fitting to canal anatomy, potentially optimizing stress distribution and load transfer characteristics [12]. However, the clinical significance of this 42.6% increase in fracture resistance must be evaluated in the context of physiological loading conditions. Normal masticatory forces in the anterior region typically range from 100-300 N, while maximum bite forces may reach 400-600 N [4]. Both post systems demonstrated fracture resistance values exceeding typical physiological loads, suggesting that the observed differences may not translate directly to clinical superiority under normal functional conditions [13, 14, 15- 16]. The higher fracture resistance of zirconia posts becomes clinically relevant in cases involving parafunctional habits, such as bruxism or clenching, where loading forces may significantly exceed normal physiological ranges. In such cases, the enhanced mechanical properties of zirconia may provide additional safety margins against catastrophic failure [17]. The contrasting failure mode patterns observed in this study represent a critical consideration for clinical decision-making. The predominance of catastrophic, non-restorable failures in the zirconia group (83.3%) reflects the material's high elastic modulus (200 GPa) and its mechanical mismatch with dentin (18 GPa) [1]. This mismatch creates stress concentration at the post-dentin interface, leading to crack initiation and propagation through the root structure. The wedge effect described in previous investigations explains the mechanism of root fracture in rigid post systems [1]. When subjected to loading, the high elastic modulus of zirconia prevents stress absorption and dissipation, instead transmitting forces directly to the surrounding dentin. This results in the development of internal stresses that exceed the fracture strength of dentin, leading to vertical root fractures [18, 19-20]. In contrast, the favorable failure pattern observed in fiber posts (73.3% restorable failures) can be attributed to their elastic modulus similarity to dentin, enabling more compatible stress distribution [1, 2]. When failures occur, they typically involve the weaker composite core material or the post-core interface, preserving the integrity of root structure and enabling successful retreatment. The results of this study support a nuanced approach to post selection that considers both mechanical requirements and failure consequences. For patients with normal occlusal function and adequate remaining tooth structure, prefabricated fiber posts may be preferred due to their favorable failure characteristics and established clinical success rates [2]. The slightly lower fracture resistance is offset by the preservation of retreatment options and reduced risk of tooth loss following failure.

Custom-milled zirconia posts may be indicated in specific clinical scenarios where maximum fracture resistance is required, such as:

[1] Patients with confirmed parafunctional habits

[2] Teeth subjected to high masticatory forces (*e.g.*, canines, premolars)

[3] Cases with minimal remaining coronal tooth structure

[4] Aesthetic zones where post translucency is critical

However, the selection of zirconia posts should be accompanied by careful consideration of long-term prognosis and acceptance of potential catastrophic failure modes.

The CAD-CAM manufacturing process used for custom-milled zirconia posts offers several theoretical advantages, including precise canal fitting and optimized post geometry [12]. However, the clinical benefits of custom fabrication versus prefabricated systems require further longitudinal investigation. The additional time, cost and technical complexity associated with custom-milled posts must be justified by demonstrable clinical advantages. Future developments in zirconia post design may address some of the observed limitations. Strategies such as surface modifications to improve bonding, geometric optimization to reduce stress concentration and hybrid designs combining rigid and flexible components may enhance clinical performance while maintaining favorable failure characteristics. Several limitations should be acknowledged in interpreting these results. The *in vitro* testing environment, while standardized, does not fully replicate the complex oral environment, including the presence of the periodontal ligament, cyclic loading patterns and biochemical factors that may influence long-term performance [15]. The static loading protocol, while standardized for comparative purposes, does not simulate the dynamic masticatory forces encountered clinically. The thermocycling protocol (5000 cycles) represents a relatively short aging period compared to the expected clinical lifespan of post restorations. Extended thermocycling and mechanical cycling protocols may reveal different performance characteristics and failure mechanisms. The use of extracted teeth introduces variability in dentin quality, canal anatomy and structural integrity that may not be representative of typical clinical cases. Patient factors such as age, medical history and oral health status significantly influence treatment outcomes but cannot be controlled in laboratory studies. Longitudinal clinical studies are essential to validate the laboratory findings and establish evidence-based guidelines for post selection. Prospective randomized controlled trials comparing custom-milled zirconia posts with established fiber post systems would provide valuable insights into long-term clinical performance, survival rates and failure patterns. Investigation of hybrid post designs that combine the mechanical advantages of zirconia with the favorable failure characteristics of fiber posts represents a promising research direction. Surface modification techniques to improve the bond strength between zirconia posts and resin cements may also enhance clinical performance. Advanced finite element analysis models incorporating patient-specific anatomy and loading conditions could provide deeper insights into stress distribution patterns and optimize post design parameters for individual clinical scenarios [17].

## Conclusion:

Custom-milled zirconia posts offer superior fracture resistance but are associated with catastrophic, non-restorable failures. Prefabricated fiber posts, despite lower strength, provide more favorable, restorable failure patterns. Hence, clinicians should select post systems based on individual case demands, balancing mechanical strength with long-term prognosis.
